# Do “Flops” Enhance Authenticity? The Impact of Influencers’ Proactive Disclosures of Failures on Product Recommendations

**DOI:** 10.3390/bs15070971

**Published:** 2025-07-17

**Authors:** Xinge Ye, Chunqing Li

**Affiliations:** School of Economics & Management, Northwest University, Xi’an 710127, China; 202110040@stumail.nwu.edu.cn

**Keywords:** influencer marketing, disclosure of failure, perceived authenticity, purchase intentions, follower scale, self-discrepancy

## Abstract

In reality, some influencers who publicly share failures can gain more attention, which defies common sense. Existing research primarily focuses on the depth of self-disclosure by influencers, which fails to explain the current phenomena. Here, we focus on negative self-disclosure and explore whether an influencer’s proactive disclosure of failures can enhance purchase intentions, along with the underlying mechanisms and boundary conditions of this strategy. We conducted three online experiments on the Credamo platform. Study 1 (N = 94) explored the main and mediating effect, whereas Study 2 (N = 238) and Study 3 (N = 238) investigated the moderating effects of observer and influencer characteristics, respectively. The following conclusions are drawn: (1) Influencers’ proactive disclosures of failures can boost purchase intentions when recommending products; (2) perceived authenticity plays a mediating role in this process; (3) the degree of viewers’ self-discrepancy moderates the mediating effect of perceived authenticity; and (4) influencers’ follower scale moderates the impact on purchase intentions. This study offers practical implications for influencers on how to enhance marketing effectiveness through self-disclosure.

## 1. Introduction

Currently, collaborating with influencers has become a crucial marketing strategy across various industries (Pan et al., 2024). As commercial messages increasingly infiltrate influencers’ organic content, consumers are no longer passive viewers but active participants (Peltier et al., 2024; C. L. Wang, 2024), and they are starting to question influencers’ authenticity (J. Zhang et al., 2023; R. Zhang et al., 2025). To address the authenticity crisis, influencers proactively disclose themselves, like personal thoughts (J. Chung et al., 2023) or private life details (Leite & de Baptista, 2022), with a particular focus on information that can enhance their image. However, seemingly negative self-disclosure has recently gained popularity on social media—deliberately sharing failures under the hashtag #flop. In fact, “flop” describes an unexpected failure, which was originally used to denote the collapse of an influencer’s persona. Since “Mianyang Culinary”—a food influencer with millions of followers—began regularly posting compilation videos of her failures and gained significant attention, others have followed suit, making “#flop” a popular content trend. Why is voluntary failure disclosure by influencers so well-received? Can this content strategy enhance product recommendation effectiveness?

This phenomenon challenges established academic paradigms that advocate for positive self-presentation by influencers (Osorio et al., 2020). In fact, existing research on influencer self-disclosure has predominantly focused on positive and in-depth disclosures, with limited exploration of negative disclosures (F. Wang & Carlson, 2024). Current theories are insufficient to explain the phenomenon. Only a few studies within traditional celebrity context have focused on this. Brooks et al. (2019) showed that entrepreneurs disclosing failures can mitigate social envy through the framing of vulnerability, and E. J. Lee et al. (2020) found that politicians sharing errors strategically boosts engagement efficacy. Owing to the clear differences between traditional celebrities and influencers (Schouten et al., 2020), as well as the new outcome variables specific to influencer marketing, we think the impact of influencers voluntarily disclosing failures on the effectiveness of a product recommendation deserves deeper exploration.

In this study, we ask the following questions: (a) Can influencers’ voluntary disclosures of failures enhance the effectiveness of product recommendations? (b) What are the mediating mechanisms underlying this effect? (c) What about the boundary conditions? We think an influencer’s proactive disclosure of failure can significantly enhance the effectiveness of a product recommendation. Given the unpredictability of human brands, we believe perceived authenticity—a defining attribute of human brands—can explain this mechanism. In addition, observers with varying self-perception patterns exhibit different interpretations of influencers’ failure disclosures (Sarial-Abi et al., 2021). Therefore, observers’ self-discrepancy levels should serve as a boundary condition. Moreover, such self-disclosure strategies are not effective for all influencers, it depends on their developmental stages (De Veirman et al., 2017), so the influencer’s follower scale should be another contextual moderator. Thus, we identify two boundary conditions to answer the previous questions: (1) the observer’s level of self-discrepancy and (2) an influencer’s follower scale.

This study employs three experiments to verify the hypotheses, yielding significant theoretical and practical contributions to influencer marketing. In terms of theoretical contributions, first, this study reveals the positive impact of an influencer’s proactive failure disclosure on influencer marketing, breaking the established paradigm that suggests influencers can only achieve effective marketing by cultivating a positive image (Osorio et al., 2020). It also expands upon the limitations of prior self-disclosure research on influencers, which often focuses solely on the depth of disclosure (F. Wang & Carlson, 2024). Second, this study uncovers the mediating role of perceived authenticity, deepening the understanding of authenticity management among influencers and revealing a counterintuitive antecedent: proactive failure disclosure thereby further enhances the comprehension of the connotation of influencer authenticity. Third, this study explores the effective conditions for implementing failure disclosure strategies from the perspectives of both observers and influencers, finding that macro-influencers applying this strategy among observers with high self-discrepancy are more conducive to product recommendations. Fourth, this study extends the traditional “pratfall effect” (Aronson et al., 1966). Unlike the traditional effect, which often involves unintentional blunders, this study focuses on influencers’ proactive failure disclosure behavior. In terms of practical implications, influencers can proactively disclose their failures on their social media profiles to enhance the recommendation effectiveness of subsequent products. However, the implementation effectiveness of this strategy depends on the influencer’s personal development stage (micro vs. macro). Additionally, influencers should align this negative self-disclosure strategy with their core audience to maximize communication impact.

## 2. Theoretical Background and Research Hypotheses

### 2.1. Influencer Self-Disclosure Strategy: Failure Disclosure

Influencer self-disclosure refers to the extent to which influencers reveal personal information. As social media platforms facilitate the widespread and frequent sharing of personal content, self-disclosure has emerged as a strategy for influencers to forge connections and influence audiences by enhancing observers’ understanding of influencers’ inner states (Ma et al., 2023; R. Zhang et al., 2025). As research in this domain advances, the study of self-disclosure has increasingly focused on specific dimensions—such as depth, breadth, frequency, and content—to elucidate their roles in audience engagement (S. S. Lee & Johnson, 2022). Among these dimensions, depth stands out as the most extensively researched (Leite & de Baptista, 2022; Klostermann et al., 2023).

Research on the thematic content of what influencers actually disclose remains relatively nascent, with increasing attention being paid to the paradoxically positive effects of negative self-disclosure. This includes the disclosure of disruptive or “damaging” content such as personal failures, sensitive information, and missteps. In the political sphere, when politicians engage with social media, their blunders or mistakes can paradoxically enhance their personal image (E. J. Lee et al., 2020). In leadership studies, leaders who disclose sensitive information are perceived as more authentic by their subordinates (L. Jiang et al., 2022). Unlike prior research, this study focuses on the phenomenon of influencers pinning “flop videos”, aiming to investigate the impact of such self-disclosure on product recommendation.

On video-centric social media platforms, video quality and visual aesthetics directly dictate audience preferences (Filieri et al., 2023). Influencers typically refine their filming and editing techniques to eliminate authentic mishaps, preserving only the most polished and seamless clips to craft a differentiated visual aesthetic experience. However, flop segments often represent behind-the-scenes bloopers that contrast with their meticulously curated content. By showcasing these bloopers as a form of self-disclosure, influencers reveal their “imperfect” selves. Pinning such “flop videos” to their personal homepages may trigger the pratfall effect (Aronson et al., 1966), wherein a minor blunder paradoxically enhances likability. This study posits that influencers who proactively display their failure clips on their profiles achieve superior product recommendation outcomes compared to those who do not. This is because influencers who openly embrace imperfections signal autonomy by prioritizing authenticity over conformity to external expectations. A key challenge in influencer marketing lies in consumers’ uncertainty about whether product endorsements stem from a genuine intrinsic motivation or commercial coercion. Consequently, influencers who voluntarily disclose failures are perceived as more credible, thereby enhancing the effectiveness of their product promotions and fostering higher purchase intentions among observers. Therefore, the following hypothesis is proposed:

**H1.**  *Influencers who proactively disclose failures on social media will generate higher purchase intentions for recommended products compared to those who do not.*

### 2.2. The Mediating Role of Perceived Authenticity

Authenticity, defined as the quality of appearing genuine, sincere, and/or truthful, is critical for influencers (Audrezet et al., 2020; Nunes et al., 2021; Vo et al., 2024). Influencer authenticity manifests in two dimensions (Chen et al., 2023): authenticity toward others, primarily through transparent sponsorship disclosures and balanced product information, and authenticity toward oneself, reflecting an intrinsic motivation and alignment between actions/words and personal values, independent of external pressures. This study focuses on the latter dimension, arguing that perceived authenticity is the key mechanism through which influencers’ proactive failure disclosures (e.g., pinning “flop videos”) influence purchase intentions.

Drawing from the Self-Determination Theory, authenticity is heightened when influencers act in accordance with their values rather than solely to please others (Kernis & Goldman, 2006). As human brands, influencers should exhibit a degree of unpredictability; disclosing “imperfect” content that may appear detrimental to their image enhances perceived authenticity (Fournier & Eckhardt, 2019). For instance, politicians who use politically incorrect language are perceived as more authentic (Rosenblum et al., 2020). Thus, we posit that overly polished influencers risk being perceived as robotic and dehumanized. In contrast, pinning “flop videos” to their profiles—rather than presenting only flawlessly edited content—significantly enhances their perceived authenticity among observers. This authenticity mitigates the skepticism often associated with sponsored content, thereby boosting purchase intentions. Therefore, the following hypothesis is proposed:

**H2.**  *Influencers who proactively disclose failures will enhance perceived authenticity among observers to a greater extent than those who do not, resulting in heightened purchase intentions.*

### 2.3. The Moderating Role of Observer Self-Discrepancy

Self-discrepancy refers to the differences and conflicts between distinct self-beliefs. The Self-Discrepancy Theory posits that individuals possess multiple selves (e.g., actual self vs. ideal self), and discrepancies between these selves generate psychological discomfort. People are motivated to reduce such discrepancies by aligning their actual self with their ideal self (Higgins, 1989). When information aligns with consumers’ goals to mitigate self-discrepancies, it becomes more persuasive and is processed more readily (Dijkstra et al., 2009).

Prior research has found that influencers’ glamorized personas exacerbate consumers’ self-discrepancies (Jaiswal et al., 2024). According to the Social Comparison Theory, influencer content on social media often represents aspirational ideals, heightening consumers’ desire for imitation (Ki & Kim, 2019). Individuals with high self-discrepancies are more likely to engage in mimicry of their favorite celebrities as a means of bridging the gap between their actual and ideal selves (Derrick et al., 2008).

We propose that consumers’ self-discrepancy levels moderate the impact of influencers’ proactive failure disclosures (e.g., pinning “flop videos”) on purchase intentions for recommended products. Self-discrepancy influences observers’ information processing styles (Sarial-Abi et al., 2021; Y. Jiang & Lei, 2025). According to Malär et al. (2018), whether an individual absorbs the information conveyed by influencers is determined by self-discrepancy. Individuals experiencing a pronounced gap between their ideal and actual selves tend to engage in more concrete information processing. Consequently, they are more attentive to influencers’ failure disclosures, and more prone to resonate with influencers (Aw & Chuah, 2021). As a result, they are more inclined to purchase products recommended by influencers who disclose failures, compared to those who do not. In contrast, individuals with low self-discrepancy, who are more satisfied with their actual selves, engage in abstract information processing; they are less susceptible to external influences, diminishing influencers’ impact, and, therefore, they may dismiss influencers’ failure disclosures as trivial, leaving their purchase decisions unaffected. Therefore, the following hypothesis is proposed:

**H3a.**  *Observers with high self-discrepancy will exhibit greater purchase intentions for products recommended by influencers who proactively disclose failures compared to those who do not.*

We further argue that consumers’ self-discrepancy levels moderate the mediating role of perceived authenticity. Influencers’ proactive failure disclosures foster a psychological proximity with audiences. Observers with high self-discrepancy, often dissatisfied with their actual selves, experience significant anxiety relief when encountering influencers who embrace “authentic imperfections” (Goldman & Kernis, 2002). This resonance enhances the influencer’s perceived authenticity, mitigating skepticism toward sponsored content and boosting purchase intentions. Conversely, observers with low self-discrepancy, who are content with their actual selves, are less susceptible to external influences. For them, influencers’ failure disclosures do not enhance perceived authenticity. Therefore, the following hypothesis is proposed:

**H3b.**  *Observers’ self-discrepancy levels will moderate the mediating effect of perceived authenticity. Specifically, higher self-discrepancy amplifies observers’ sensitivity to influencers’ proactive failure disclosures, leading to greater perceived authenticity and, consequently, higher purchase intentions, compared to observers with low self-discrepancy.*

### 2.4. The Moderating Role of Influencer Type

In influencer marketing research, the number of followers serves as a critical criterion for categorizing influencers (Conde & Casais, 2023). The distinction between micro-influencers (1000–100,000 followers) and macro-influencers (100,000–1,000,000 followers) hinges on their potential reach. Prior studies have explored differences in the relationship that micro- and macro-influencers build with their followers (Wies et al., 2023).

When it comes to product endorsements, scholars generally argue that macro-influencers are perceived as less credible than micro-influencers. Despite their popularity and status as opinion leaders (De Veirman et al., 2017), macro-influencers’ frequent exposure to commercial sponsorships and monetization opportunities often triggers consumer skepticism (Steils et al., 2022). Conversely, micro-influencers, with smaller follower bases, cultivate closer relationships with their audiences, enhancing their perceived credibility.

This study posits that the impact of influencers’ proactive failure disclosures (e.g., pinning “flop videos”) on purchase intentions varies based on the follower scale, with macro-influencers benefiting more. Previous research suggests that the effectiveness of failure disclosures depends on the discloser’s baseline status and competence. Aronson et al. (1966) demonstrated that a nearly flawless individual who occasionally errs or “fails” is perceived as more human, whereas the same mistake made by an average person elicits minimal impact. Thus, when macro-influencers with large followings pin “flop videos” on their profiles, the contrast between their idealized image and disclosed vulnerability amplifies psychological proximity with observers, boosting purchase intentions for recommended products. In contrast, micro-influencers employing this strategy are unlikely to see significant effects, as failure is perceived as more routine for emerging influencers. Therefore, the following hypothesis is proposed:

**H4a.**  *Macro-influencers who proactively disclose failures on social media will elicit greater purchase intentions for recommended products compared to micro-influencers.*

We further argue that the impact of failure disclosures on perceived authenticity differs between macro- and micro-influencers. Competent individuals who “stumble” are perceived as more relatable (Helmreich et al., 1970). As established personal brands and opinion leaders, macro-influencers create stronger contrast effects when disclosing failures, thereby enhancing their perceived authenticity among observers. In contrast, micro-influencers, still in the developmental stages, are perceived as experiencing failure more frequently; thus, proactive disclosures do not significantly elevate their authenticity. Therefore, the following hypothesis is proposed:

**H4b.**  *The influencer’s follower scale will moderate the mediating role of perceived authenticity. Specifically, macro-influencers who proactively disclose failures on social media will enhance perceived authenticity to a greater extent than micro-influencers, thereby boosting purchase intentions for recommended products.*

**Figure 1:**
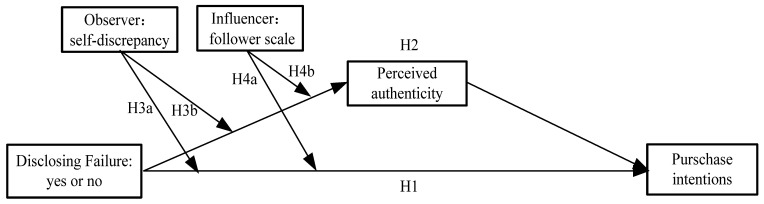
Research model.

The research model diagram presented in this paper is illustrated in Figure 1.

## 3. Methodology

This study aims to explore the impact of influencers’ proactive failure disclosures on product recommendation effectiveness, as well as the underlying mechanisms and boundary conditions. To better examine the causal relationships among variables, we employed an experimental research method. Regarding the phenomenon under investigation, it is prevalent among skill-based influencers, particularly in the culinary domain, and we conducted three experiments within the context of food influencers to test all the hypotheses (see Table 1 for details).

In Study 1, we utilized the personal homepage of a real influencer with moderate popularity (“Miao Er Ge”) as the experimental material to test the main effect (H1) and the mediating effect (H2). We demonstrated that influencers’ proactive failure disclosures can positively affect product recommendation effectiveness, and perceived authenticity plays a mediating role in this process. In Study 2, we replaced the experimental material and selected another real influencer with moderate popularity (“AWan”) to test H1, H2, and the moderating role of observer self-discrepancy on the main effect (H3a) and the mediating effect (H3b). The results revealed that the level of the observer’s self-discrepancy can significantly moderate the main effect and the mediating effect of perceived authenticity. In Study 3, we again replaced the experimental material in a virtual influencer (“dailydrunk”) scenario to test H1, H2, and the moderating role of an influencer’s follower scale on the main effect (H4a) and the mediating effect (H4b). The study found that the influencer’s follower scale can significantly moderate the main effect but does not moderate the mediating effect of perceived authenticity. All experiments were completed from 3 January to 23 February 2025.

The reasons for the selection of participants, social media platform, and data collection methods in this study are as follows: given that “flop” is a recently emerged internet buzzword and younger generations are more acquainted with this phenomenon, we restricted our study participants to those under 30 years old. Little Red Book is one of the most popular applications in China with over 300 million daily active young users; we selected it as the representative social media platform for hypothesis testing. To ensure the diversity and validity of data sources, we recruited participants through the Credamo platform, a survey hosting tool that has a national sample pool of over 3 million participants.

## 4. Study 1

### 4.1. Design

The objective of Study 1 was to examine the main and mediating effects of perceived authenticity; therefore, we used a single-factor between-groups design (failure disclosure vs. no failure disclosure). We manipulated the independent variable by placing or not placing a “flop video” on the influencer’s homepage. To simulate a realistic scenario, materials were sourced from the Little Red Book profile of a real influencer (called “Miao Er Ge”).

We recruited 100 participants (under 30 years old) through the Credamo platform, selecting participants who demonstrated high-quality answers and possessed good credibility (with an adoption rate of 80%) as our recruitment targets. To ensure the quality of their responses, we included systematic screening questions in the questionnaire. Additionally, we excluded participants who completed the survey in an excessively short or long time period, as well as those who did not frequently use social media. Ultimately, we obtained 94 valid participants as the final sample. These participants (N = 94) were randomly assigned to a single-factor (failure disclosure vs. no failure disclosure) between-subjects design (N_failure present_ = 47, N_failure absent_ = 47; 51.1% female, M_age_ = 24.8 years; and 8.5% bachelor’s degree, 76.6% master’s degree, and 14.9% doctoral degree). After all participants’ questionnaires were approved, they received corresponding compensation (5 RMB) issued by the Credamo platform.

### 4.2. Procedure

**Figure A1:**
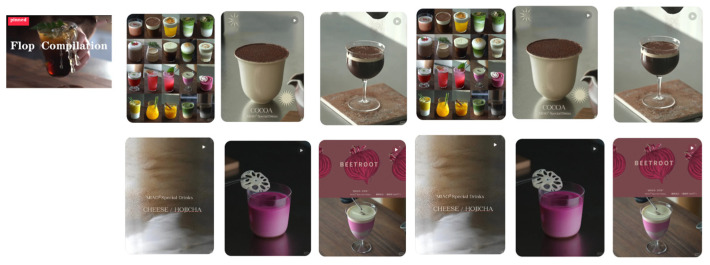
Manipulation of disclosure for Study 1. Reproduced with permission from “Miao Erge”; this is the homepage of the influencer. https://www.xiaohongshu.com/user/profile/5bde5b96852a0c00010ec343?xsec_token=ABnlestrtf4J8CxRYeXa6MXoUvcG5C3KlgB-pvucSDAOg%3D&xsec_source=pc_search, accessed on 10 July 2025.

Participants were first introduced to a scenario: “Imagine you follow a culinary influencer on Little Red Book. Curious, you visit their profile page”. Then, they were randomly assigned to either the failure-present group or the failure-absent group (Web Appendix A, Figure A1). In the failure-absent group, participants viewed six standard food preparation video covers on the influencer’s profile page, followed by a pre-recorded one-minute conventional video on the subsequent page. In the failure-present group, in addition to the six standard covers, participants saw a pinned “failure compilation” video cover at the top of the profile page. On the subsequent page, in addition to the conventional video, they watched an additional failure video. After the independent variable manipulation, we conducted a manipulation check to confirm experimental conditions (e.g., “Did the influencer disclose a failure in their videos?”) and measured dependent variables (e.g., purchase intentions), mediating variables (e.g., perceived authenticity), alternative explanatory variables (attractiveness and trustworthiness), and demographic information (e.g., age, gender, and education level).

### 4.3. Measures

Perceived authenticity was measured by Wood et al.’s (2008) four-item scale (Cronbach’s α = 0.806). Sample items included “This influencer manages their social media account in a sincere manner” and “This influencer prioritizes being authentic over seeking popularity.” The dependent variable was the purchase intentions (Zeithaml, 1988; Cronbach’s α = 0.895), operationalized as “To what extent would you be willing to buy the product recommended by this influencer?”; attractiveness (Cronbach’s α = 0.801); and trustworthiness (Cronbach’s α = 0.832) (Munnukka et al., 2016).

### 4.4. Results

Manipulation Check: Prior to the main experiment, a manipulation check was conducted to validate the placement of failure videos on the influencer’s profile page. In total, 64 university students evaluated the experimental stimuli, answering: “Did the influencer pin a ‘failure compilation’ video on their profile?” In the failure-present condition, 98% of participants correctly identified the pinned failure video. In the failure-absent condition, 100% of participants correctly identified the absence of a failure video. This confirmed the successful manipulation of the independent variable.

Purchase Intentions: A one-way ANOVA examined the impact of failure disclosure on purchase intentions. The results revealed a significant difference between the two groups (M_present_ = 5.47, SD = 1.18, M_absent_ = 5.25, SD = 1.14, F(1, 93) = 4.75, and *p* < 0.05). Thus, H1 was supported.

Perceived Authenticity: A one-way ANOVA assessed the effect of failure disclosure on perceived authenticity. The results showed a significant difference between the two groups (M_present_ = 5.33, SD = 0.93, M_absent_ = 4.51, SD = 1.06, F(1, 93) = 13.98, and *p* < 0.001).

Mediation Analysis: Using PROCESS Model 4, we tested the mediating role of perceived authenticity in the relationship between failure disclosure and purchase intentions, controlling for demographic variables (gender, age, and education; refer to Table 2). First, we explored the direct effect of influencer failure disclosure on purchase intentions (b = −0.0002, 95% CI [−0.382, 0.382] (includes 0)). Second, we explored the moderated mediation effect. The results indicated the indirect effect was significant (b = −0.476, 95% CI [−0.824, −0.242] (excluding 0)). Thus, H2 was supported.

Alternative Explanations: To rule out confounding effects, we tested perceived attractiveness and trust. No significant difference was observed for attractiveness (M_present_ = 5.44, SD = 1.32; M_absent_ = 5.53, SD = 0.96, F(1, 93) = 0.194, and *p* = 0.660). No significant difference was observed for trust (M_present_ = 5.33, SD = 0.95, M_absent_ = 5.29, SD = 1.08, F(1, 93) = 0.001, and *p* = 0.971). Therefore, alternative explanations (attractiveness and trust) were ruled out, strengthening the validity of the mediating role of perceived authenticity.

### 4.5. Discussion

In Study 1, we found that an influencer’s voluntary failure disclosure significantly enhances purchase intentions (H1 was supported), and perceived authenticity mediates the relationship (H2 was supported). Meanwhile, we have also excluded alternative explanations regarding attractiveness and credibility. However, Study 1 only examined the main effects and mediating effects under a single experimental material. Next, in Study 2, we will repeat the tests of H1 and H2 using different experimental materials and explore the moderating role of observers’ self-discrepancy levels.

## 5. Study 2

### 5.1. Design

The objective of Study 2 was to examine the moderating role of observers’ self-discrepancy levels on the mediating effect of perceived authenticity. Given that the degree of self-discrepancy is a personal characteristic with a certain level of stability (Higgins, 1989), we did not manipulate the observers’ degree of self-discrepancy; instead, we measured it directly. Therefore, we also employed a single-factor between-groups design (failure disclosure vs. no failure disclosure). To simulate a realistic scenario, we also used a real influencer’s profile (called “Awan”) from Little Red Book.

We recruited 245 participants (under 30 years old) through the Credamo platform. To ensure the quality of their responses, we included systematic screening questions in the questionnaire. Additionally, we excluded participants who completed the survey in an excessively short or long time period, as well as those who did not frequently use social media. Ultimately, we obtained 238 valid participants as the final sample, which were randomly assigned to a single-factor design (present vs. absent) (N_present_ = 120, N_absent_ = 118; 53.8% female, M_age_ = 24.24 years; and 12.2% bachelor’s degree, 72.7% master’s degree, and 14.3% doctoral degree). After all participants’ questionnaires were approved, they received corresponding compensation (5 RMB) issued by the Credamo platform.

### 5.2. Procedure

**Figure A2:**
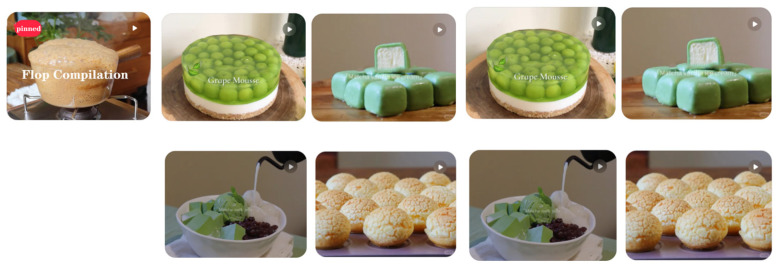
Manipulation of disclosure for Study 2. Reproduced with permission from “AWan”; this is the homepage of the influencer. https://www.xiaohongshu.com/user/profile/5d326cd40000000012005904?xsec_token=ABx0nOfYxB14G9u5yyoORJVc1575H38HFCcnW4JGhRHTY%3D&xsec_source=pc_search, accessed on 10 July 2025.

**Figure A3:**
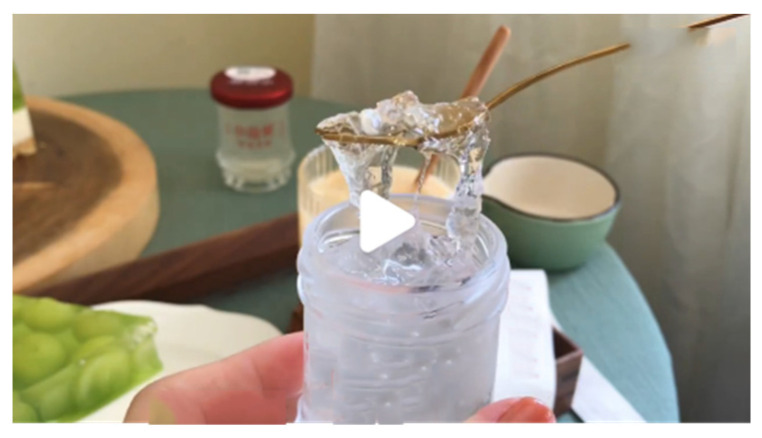
Screenshot of product placement used in Study 2. Reproduced with permission from “AWan”; the screenshot is from this influencer’s video. https://www.xiaohongshu.com/discovery/item/5f1d62a5000000000100108b?source=webshare&xhsshare=pc_web&xsec_token=ABryUCZxf3HU562B0K0ObTZmnJqEYH_iafULJTdRbJdX0=&xsec_source=pc_share, accessed on 10 July 2025.

As in Study 1, participants were randomly assigned to either the failure-present group or the failure-absent group (Web Appendix A, Figure A2). In the failure-absent group, participants viewed four standard food preparation video covers on the influencer’s profile page, followed by a pre-recorded one-minute conventional video on the subsequent page. In the failure-present group, in addition to the four standard covers, participants saw a pinned “failure compilation” video cover at the top of the profile page. On the subsequent page, they watched two videos: first, the pre-recorded conventional video, followed by an additional failure video. In order to simulate a sponsored scenario in a real-world context, participants were then shown a screenshot of a sponsored product embedded within a conventional video (Web Appendix A, Figure A3), simulating a realistic product recommendation context. After the independent variable manipulation, we conducted a manipulation check to confirm the experimental conditions (e.g., “Did the influencer disclose a failure in their videos?”) and measured the dependent (purchase intentions), mediating (perceived authenticity), and moderating variables (self-discrepancy), as well as demographic information (e.g., age, gender, and education level).

### 5.3. Measures

Perceived authenticity was measured using a validated scale as noted in Study 1 (Cronbach’s α = 0.797). Purchase intentions were assessed using a separate scale (Cronbach’s α = 0.876). Self-discrepancy was operationalized as the absolute difference between the ideal-self and actual-self scores (Aw & Chuah, 2021). In this study, we focused on influencers voluntarily showcasing their failures, which is highly correlated with competence, so we selected measurement items related to personal competence to test self-discrepancy (Z. Wang et al., 2017) (Cronbach’s α = 0.895). Sample items included “To what extent is your ideal self competent, skillful, capable, and intelligent?” and “To what extent is your actual self competent, skillful, capable, and intelligent?”

### 5.4. Results

Manipulation Check: Consistent with Study 1, participants responded to a dichotomous question about the experimental stimuli: “Did the influencer display a ‘failure compilation’ video on their profile page?” In the failure-present condition, 100% of participants correctly identified the pinned failure video. In the failure-absent condition, 100% of participants correctly identified the absence of a failure video. This confirmed the successful manipulation of the independent variable.

Purchase Intentions: A one-way ANOVA examined the impact of failure disclosure on purchase intentions. The results revealed a significant difference (M_present_ = 5.22, SD = 0.82; M_absent_ = 4.92, SD = 1.12, F(1, 237) = 4.96, and *p* < 0.05). Thus, H1 was supported. Failure disclosure increased purchase intentions.

Mediation Analysis: Using PROCESS Model 4, we tested the mediating role of perceived authenticity in the relationship between failure disclosure and purchase intentions, controlling for demographic variables (gender, age, and education; refer to Table 3). The results indicated that the direct effect is not significant (b = 0.185, 95% CI [−0.048, 0.419] (includes 0)). The indirect effect is significant (b = −0.458, 95% CI [−0.704, −0.274] (excludes 0)). Perceived authenticity completely mediated the effect of failure disclosure on purchase intentions; thus, H2 was supported.

**Figure 2:**
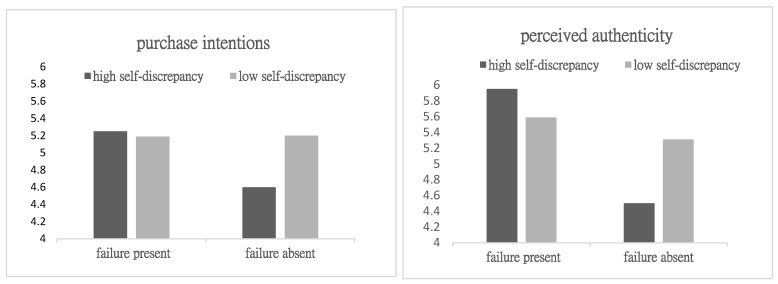
The interaction of failure disclosure and self-discrepancy in Study 2.

Two-way ANOVA on Purchase Intentions: A 2 (failure disclosure: yes vs. no) × 2 (self-discrepancy: high vs. low) factorial ANOVA was conducted using purchase intentions as the dependent variable. The results revealed a significant interaction effect (F(1, 237) = 7.25, *p* < 0.01). Further analysis of simple effects revealed that the high self-discrepancy group showed a significant difference (M_present_ = 5.25, SD = 0.93; M_absent_ = 4.60, SD = 1.18, F(1, 237) = 12.447, and *p* < 0.01) (Figure 2). However, no significant difference was noted in the low self-discrepancy group (M_present_ = 5.19, SD = 0.69; M_absent_ = 5.20, SD = 1.10, F(1, 237) = 0.014, and *p* = 0.905). Thus, H3a was supported. The effect of failure disclosure on purchase intentions was moderated by self-discrepancy, with a stronger positive effect observed among high self-discrepancy observers.

Two-way ANOVA on Perceived Authenticity. A 2 (failure disclosure: yes vs. no) × 2 (observer self-discrepancy: high vs. low) factorial ANOVA was conducted using perceived authenticity as the dependent variable. The results revealed a significant interaction effect between failure disclosure and observer self-discrepancy (F(1, 237) = 24.57, *p* < 0.001). Further analysis of the simple effects revealed that in the high self-discrepancy observer group, the effect of failure disclosure on perceived authenticity was significant (M_resent_ = 5.95, SD = 0.706; M_absent_ = 4.50, SD = 1.14, F(1, 237) = 66.48, and *p* < 0.001) (Figure 2). In the low self-discrepancy observer group, the effect of failure disclosure on perceived authenticity was not significant (M_present_ = 5.59, SD = 0.85; M_absent_= 5.31, SD = 0.79, F(1, 237) = 2.63, and *p* = 0.106).

Moderated Mediation Effect: We tested the moderated mediation model using PROCESS Model 7, employing 5000 bootstrap resamples and 95% confidence intervals (CIs) to assess the research framework (refer to Table 4). First, we explored the direct effect of influencer failure disclosure on purchase intentions (b = 0.185, 95% CI [−0.048, 0.418] (includes 0, non-significant)). Second, we explored the moderated mediation effect (index of the conditional indirect effect; b = 0.679, 95% CI [0.382, 1.041] (excludes 0, significant)). In further analysis, we found that in high self-discrepancy observers, the indirect effect via perceived authenticity was significant (b = −0.829, 95% CI [−1.184, −0.528] (excludes 0)). However, in low self-discrepancy observers, the indirect effect via perceived authenticity was not significant (b = −0.150, 95% CI [−0.346, 0.016] (includes 0)). H3b was supported. The mediating role of perceived authenticity in the relationship between failure disclosure and purchase intentions was moderated by observer self-discrepancy, with a significant effect observed only among low self-discrepancy observers.

### 5.5. Discussion

In Study 2, we replicated the conclusions of Study 1 (H1 and H2), thereby enhancing the robustness of the results. More importantly, we explored the boundary conditions from observer perspectives by examining the level of self-discrepancy. The results show that an observer’s self-discrepancy moderates both the main effect of failure disclosure on purchase intentions (H3a was supported) and the mediating role of perceived authenticity (H3b was supported). Specifically, high self-discrepancy observers attribute voluntary failure disclosure to authenticity, amplifying downstream marketing effectiveness. Conversely, low self-discrepancy observers show no such response. However, Study 2 only examined the moderating variables from the observer’s perspective and did not focus on the characteristics of the influencers. We will conduct a further examination in Study 3.

## 6. Study 3

### 6.1. Design

The purpose of Study 3 was to investigate the moderating role of influencer follower scale on the effect of failure disclosure; therefore, a 2 (failure disclosure: yes vs. no) × 2 (follower scale: macro vs. micro) between-subjects design was employed. We manipulated “follower scale” by presenting participants with the personal homepage of influencers displaying their follower counts; to eliminate the confounding effects of influencer familiarity, we used a fictional influencer account (“dailydrunk”).

We recruited 250 participants (under 30 years old) through the Credamo platform. To ensure the quality of their responses, we included systematic screening questions in the questionnaire. Additionally, we excluded participants who completed the survey in an excessively short or long time period, as well as those who did not frequently use social media. Ultimately, we obtained 238 valid participants as the final sample, with participants randomly assigned to a 2 (failure disclosure: yes vs. no) × 2 (follower scale: macro vs. micro) between-subjects design (N_present+Macro_ = 60, N_absent+Macro_ = 59, N_present+Micro_ = 59, and N_absent+Micro_ = 60; 55.9% female, M_Age_ = 24.2; and 9.7% bachelor’s degree, 74.4% master’s degree, and 15.1% doctoral degree). After all participants’ questionnaires were approved, they received corresponding compensation (5 RMB) issued by the Credamo platform.

### 6.2. Procedure

**Figure A4:**
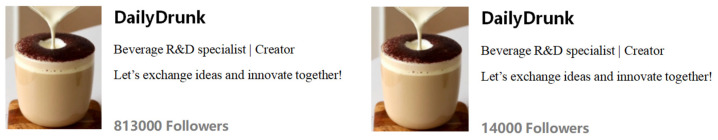
Manipulation of influencer type in Study 3.

**Figure A5:**
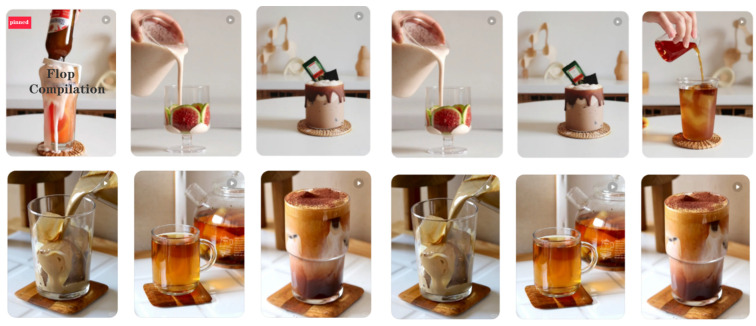
Manipulation of disclosure in Study 3. https://www.xiaohongshu.com/user/profile/5b6c47555d3f3f000174102f?xsec_token=ABjkogxWRt_6ny8rBk1EYqAsd_fnI3mlYPnUelY1hJinw=&xsec_source=pc_search, accessed on 10 July 2025.

**Figure A6:**
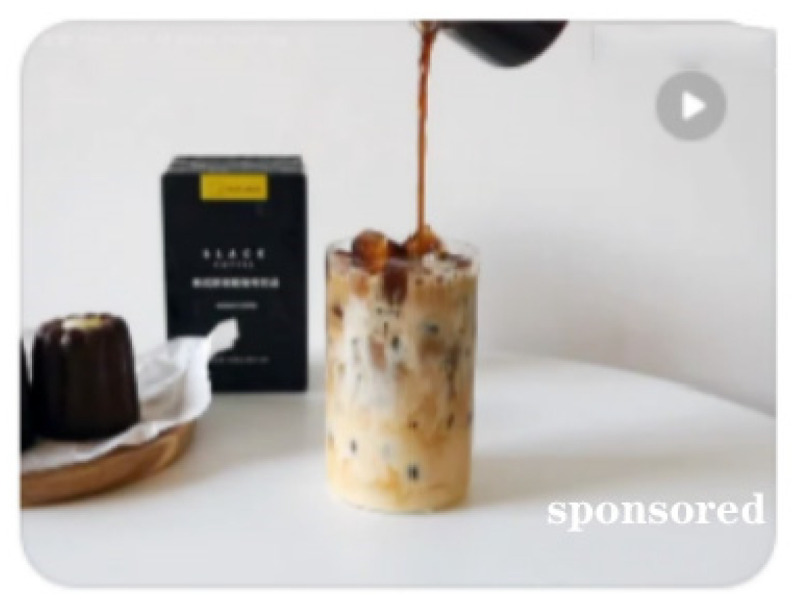
Sponsored posts in Study 3.

Participants were randomly assigned to one of four groups, following a 2 (failure disclosure: yes vs. no) × 2 (follower scale: macro vs. micro) design. They were first introduced to a scenario: “Imagine you follow a culinary influencer on Little Red Book. Curious, you visit their profile page”. Then, they viewed the homepage of the fictional influencer “dailydrunk” (Web Appendix A, Figure A4); in the macro-influencer group, the influencer had 813,000 followers, while in the micro-influencer group, they had 14,000 followers. The procedures and failure disclosure manipulation are the same as described in Studies 1 and 2 (Web Appendix A; Figure A5). In order to simulate a sponsored scenario in a real-world context, participants were then shown a sponsored video cover (labeled as such) on the influencer’s homepage (Web Appendix A, Figure A6). Afterward, participants completed the manipulation checks and measures of key variables.

### 6.3. Measures

Perceived authenticity (Cronbach’s α = 0.783) and purchase intentions (Cronbach’s α = 0.876) were measured as noted in Study 1.

### 6.4. Results

Manipulation Checks: As noted in Studies 1 and 2, participants responded to true/false questions about the experimental stimuli, and 100% correctly identified whether the food influencer’s homepage featured a failure video (yes/no). Thus, failure disclosure manipulation was successful. In addition, 100% correctly identified the influencer’s follower scale tier (macro vs. micro), so the follower scale manipulation also succeeded.

Purchase Intentions: We conducted a one-way ANOVA with failure disclosure (yes vs. no) as the independent variable and purchase intentions as the dependent variable. The results revealed a significant difference between the two groups (M_present_ = 5.15, SD = 1.17; M_absent_ = 4.88, SD = 1.27, F(1, 237) = 5.191, and *p* < 0.05). Thus, failure disclosure positively affects purchase intentions; H1 was supported.

Mediation Analysis: We tested the mediating role of perceived authenticity in the relationship between failure disclosure and purchase intentions, controlling for demographic variables (gender, age, and education) (Table 5). The results indicated that the direct effect was not significant (b = 0.292, 95% CI [−0.029, 0.613] (includes 0)). The indirect effect was significant (b = −0.674, 95% CI [−0.909, −0.470] (excludes 0)). Perceived authenticity fully mediated the effect of failure disclosure on purchase intentions, thus supporting H2.

**Figure 3:**
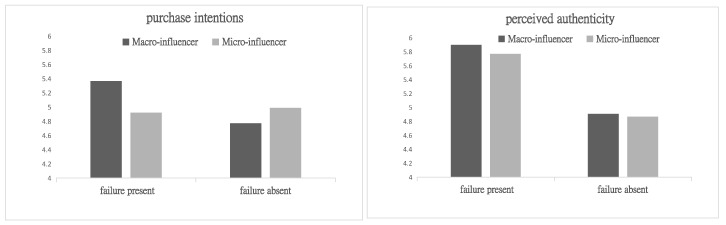
The interaction of failure disclosure and follower scale in Study 3.

Two-way ANOVA on Purchase Intentions (Failure Disclosure × Follower Scale): A 2 (failure disclosure: yes vs. no) × 2 (follower scale: macro vs. micro) factorial ANOVA was conducted. The interaction effect was significant (F(1, 237) = 9.43, *p* < 0.01). Further analysis of the simple effects revealed significant results in the macro-influencer group (M_present_ = 5.37, SD = 1.07; M_absent_ = 4.77, SD = 1.36, F(1, 237) = 14.45, and *p* < 0.001) (Figure 3). However, the results were not significant for the micro-influencer group (M_present_ = 4.92, SD = 1.23; M_absent_ = 4.99, SD = 1.18, F(1, 237) = 0.071, and *p* = 0.791). In conclusion, for macro-influencers, disclosing failure videos significantly increased purchase intentions. For micro-influencers, failure disclosure had no significant effect on purchase intentions. Thus, H4a was supported.

Two-way ANOVA on Perceived Authenticity: A 2 (failure disclosure: yes vs. no) × 2 (follower scale: macro vs. micro) factorial ANOVA was conducted with perceived authenticity as the dependent variable. The main effect of failure disclosure was significant (F(1, 237) = 62.91, *p* < 0.001). The interaction effect between failure disclosure and follower scale was not significant (F(1, 237) = 1.608, *p* = 0.206). Further analysis of simple effects revealed that the result was significant in the macro-influencer group (M_present_ = 5.90, SD = 0.78; M_absent_ = 4.91, SD = 1.01, F(1, 237) = 36.50, and *p* < 0.001) (Figure 3). In the micro-influencer group, the result was also significant (M_present_ = 5.77, SD = 0.85; M_absent_ = 4.87, SD = 1.22, F(1, 237) = 26.50, and *p* < 0.001). In conclusion, failure disclosure significantly enhanced perceived authenticity for both the macro-influencer and micro-influencer groups. The interaction effect was non-significant, indicating that the effect of failure disclosure on perceived authenticity did not differ between macro- and micro-influencers.

Moderated Mediation Effect: We examined the moderated mediation model using PROCESS Model 7, employing 5000 bootstrap resamples and 95% confidence intervals (CIs) to test the research framework. The direct effect of influencer failure disclosure on purchase intentions was not significant (b = 0.292, 95% CI [−0.029, 0.613] (includes 0)). The moderated mediation effect was significant (index of conditional indirect effect; b = 0.219, 95% CI [−0.081, 0.581] (includes 0)). In further analysis (Table 6), we found that in the macro-influencer group, the indirect effect via perceived authenticity was not significant (b = −0.798, 95% CI [−1.144, −0.532] (excludes 0)). However, in the micro-influencer group, the indirect effect via perceived authenticity was significant (b = −0.579, 95% CI [−0.856, −0.360] (excludes 0)). In conclusion, the moderating role of influencer follower number on the indirect effect of failure disclosure on purchase intentions via perceived authenticity was not confirmed. Therefore, H4b was not supported.

### 6.5. Discussion

In Study 3, we also replicated the conclusions of Study 1 (H1 and H2), thereby enhancing the robustness of the results. More importantly, we found that follower scale significantly moderated the direct effect of failure disclosure on purchase intentions (H4a was supported). Specifically, macro-influencers’ failure disclosure significantly increased purchase intentions, whereas micro-influencers’ use of this strategy yielded negligible effects. However, influencer follower scale did not moderate the mediating effect of perceived authenticity (H4b was not supported). This result does not align with our hypothesis, and we attempted to explore the underlying significance behind the data findings (C. L. Wang, 2025a, 2025b).

We attribute this null finding to two factors: first, laboratory-induced constraints. In our hypothesis, we posit that the key reason why an influencer’s follower scale can moderate the mediating effect of perceived authenticity lies in the different psychological expectations observers have for influencers with varying follower scales. However, our laboratory setting may not be able to fully recreate a real-world scenario. Observers may not internalize status differences between micro/macro-influencers when follower scales are merely displayed (vs. organically experienced), preventing expectation violation mechanisms from activating. Based on this, alternative influencer characteristics—such as role type (entertainment vs. functionality oriented) or brand positioning (warmth vs. competence)—may supersede follower scale in authenticity mediation.

Second, it is important to recognize a fundamental distinction in how an influencer’s proactive failure disclosure affects two key outcomes: perceived authenticity and purchase intentions. When it comes to perceived authenticity (impression formation), observers engage in central processing, scrutinizing the disclosure content itself without retrieving peripheral cues (e.g., follower scale). When it comes to purchase intentions (behavioral outcome), commercial signals trigger peripheral processing, where observers rapidly retrieve contextual heuristics (e.g., follower scale) as judgmental cues (Shen et al., 2025). In this study, the influencers’ self-disclosure strategies and commercial information are presented separately in our study. It is precisely the difference between interpersonal impression outcomes and marketing outcomes that explains why the follower scale moderated purchase intentions (H4a) but not authenticity (H4b). More importantly, it reveals a new question—when product placement is embedded within failure disclosures, can the follower scale moderate perceived authenticity?

In conclusion, Study 3 partially supports Hypothesis 4 and also provides us with a profound understanding of the differences between influencers’ perceived images and consumers’ purchase intentions.

## 7. General Discussion

### 7.1. Key Findings

This study investigated how influencers’ voluntary failure disclosures affect product recommendation effectiveness on social media, as well as the underlying mediating mechanisms and boundary conditions through three experiments. We found that influencers’ voluntary failure disclosures significantly enhance purchase intentions, and perceived authenticity is the mediator. More importantly, we explored the boundary conditions from observer and influencer perspectives. The results show that an observer’s self-discrepancy moderates both the main effect of failure disclosure on purchase intentions and the mediating role of perceived authenticity. Follower scale significantly moderated the direct effect of failure disclosure on purchase intentions but did not moderate the mediating effect of perceived authenticity. We attribute the invalid result to two factors: laboratory-induced constraints, and the essential distinction between the impression of an influencer and purchase intentions. All these conclusions offer certain theoretical contributions and practical implications for the research on influencer marketing.

### 7.2. Theoretical Implications

First, this study reveals the positive impact of influencers’ proactive failure disclosures on influencer marketing, breaking the existing research paradigm that suggests influencers can only achieve effective marketing by cultivating a positive image (Osorio et al., 2020). It also expands the limitations of prior self-disclosure research on influencers, which often focuses solely on the depth of disclosure (F. Wang & Carlson, 2024) and enriches the strategies for influencer marketing.

Second, this study uncovers the mediating role of perceived authenticity, deepening our understanding of authenticity management among influencers. While previous research primarily examines how in-depth disclosure enhances authenticity (S. Chung & Cho, 2017; Leite & de Baptista, 2022), this study reveals a counterintuitive antecedent: proactive failure disclosure—a negative behavior—that aligns with Fournier and Eckhardt’s (2019) discussion of the “unpredictability” of human brands, thereby enriching the connotation of influencer authenticity.

Third, this study addresses the question of which types of influencers are more effective in employing this strategy and among which audience segments (Brooks et al., 2019; E. J. Lee et al., 2020). Macro-influencers achieve more favorable product recommendations when employing this strategy among observers with high self-discrepancy. Interestingly, we find that regardless of whether influencers have a large or small follower base, failure disclosure significantly influences perceived authenticity. This conclusion implies that the presence of commercial cues determines whether observers take peripheral information into account when evaluating perceived authenticity.

Fourth, this study extends the traditional “pratfall effect” (Aronson et al., 1966). While the classic effect typically involves unintentional blunders, this research focuses on an influencer’s intentional failure disclosure. The findings reveal that perceived authenticity, rather than perceived attractiveness, serves as the core mediating variable, with an observer’s self-discrepancy acting as a moderator—highlighting fundamental differences from the traditional “pratfall effect.”

### 7.3. Practical Implications

First, influencers should be brave to show failures. Influencers always face the dual challenge of cultivating genuine connections with followers while maintaining an elegant public persona; the conclusions of this study inspire influencers to not be obsessed with creating a flawless personal image. Instead, boldly revealing their shortcomings can actually enhance marketing outcomes. Second, influencers should decide whether to adopt a failure disclosure strategy based on their own growth stage. Not all influencers can benefit from the strategy of disclosing failures. Macro-influencers can substantially enhance product recommendation outcomes by strategically showcasing their failures, whereas micro-influencers cannot. Influencers should also tailor the failure disclosure strategy to the target audience. Not all observers are willing to pay the price for mistakes. Only observers with a high self-discrepancy exhibit significantly higher purchase intentions toward products endorsed by influencers who proactively disclose failures. Third, when selecting influencer partners, brands should not solely favor those with a flawless public persona but should also pay attention to influencers who are willing to showcase their imperfections, as this might bring unexpected surprises. Moreover, this pleasant surprise is not limited to the form of sponsorship.

### 7.4. Limitations and Future Directions for Research

First, our experimental approach ensures a strong internal validity but has a reduced external validity. The experimental scenarios may not reflect real-world influencer practices with varying follower scales. Future research should combine field experiments with platform analytics data and verify hypotheses using a mixed-methods approach (C. L. Wang, 2025a). Second, future studies should explore additional boundary conditions, this study examined observer and influencer characteristics as boundary conditions. Future research could investigate platform-specific factors (e.g., pinning position of failure videos, thumbnail design, and call-to-action messaging), as some influencers opt to pin non-failure content instead. Third, this study focused solely on positive mediating effects (e.g., perceived authenticity). Future work should explore negative outcomes (e.g., perceived manipulativeness) and their mediators. Open-ended interviews revealed that some audiences perceive staged failure videos as inauthentic; thus, future studies could compare self-deprecating vs. self-promotional failure narratives.

## 8. Conclusions

Influencers often engage in personal image-building through positive self-disclosure. However, a new counterintuitive strategy has emerged on social media. Specifically, influencers pin their failure clips at the top of their social media platforms. To date, it remains unclear whether this negative self-disclosure by influencers is effective in promoting product recommendations, and what mechanisms account for this impact. In this study, we explored the impact of influencers’ voluntary disclosures of failures on the effectiveness of product recommendations, as well as the mediating mechanisms and boundary conditions. The results demonstrate that influencers’ voluntary disclosures of failures can enhance perceived authenticity, thereby effectively promoting purchase intentions for recommended products. However, this strategy is not always effective; its efficacy is influenced by influencer scale and observer self-discrepancy. High self-discrepancy observers exhibit stronger purchase intentions, while low-discrepancy individuals remain unaffected. Macro-influencers significantly boost recommendations through failure disclosure, whereas micro-influencers gain no meaningful advantage. Thus, the answer to whether failure disclosure improves recommendation efficacy is conditionally affirmative—contingent on both influencer reach and audience psychology.

## Figures and Tables

**Table 1:** Overview of the method and hypotheses (for all studies).

	Experimental Situation	Experimental Purpose	Hypotheses Tested	Results
Study 1 (N = 94)	Real influencer (“Miao Er Ge”)	Main effect Mediating effect	H1 H2	Yes Yes
Study 2 (N = 238)	Real influencer (“AWan”)	Moderating effect (observer self-discrepancy)	H1 H2 H3a H3b	Yes Yes Yes Yes
Study 3 (N= 238)	Fictional influencer (“dailydrunk”)	Moderating effect (influencer follower scale)	H1 H2 H4a H4b	Yes Yes Yes No

**Table 2:** Mediation test (Study 1).

	Bootstrapping		BC 95% CI	
	β	SE	Lower Bound	Upper Bound
Indirect effect	−0.476	0.014	−0.824	−0.242
Direct effect	−0.0002	0.019	−0.382	0.382

**Table 3:** Mediation test (Study 2).

	Bootstrapping		BC 95% CI	
	β	SE	Lower Bound	Upper Bound
Indirect effect	0.458	0.010	−0.704	−0.274
Direct effect	0.185	0.012	−0.048	0.419

**Table 4:** Moderated mediation effect test (Study 2).

	Bootstrapping		BC 95% CI	
	β	SE	Lower Bound	Upper Bound
High self-discrepancy	−0.829	0.016	−1.184	−0.528
Low self-discrepancy	−0.150	0.013	−0.346	0.016

**Table 5:** Mediation test (Study 3).

	Bootstrapping		BC 95% CI	
	β	SE	Lower Bound	Upper Bound
Indirect effect	−0.674	0.011	−0.909	−0.470
Direct effect	0.292	0.016	−0.029	0.613

**Table 6:** Moderated mediation effect test (Study 3).

	Bootstrapping		BC 95% CI	
	β	SE	Lower Bound	Upper Bound
Macro-influencer	−0.798	0.016	−1.144	−0.532
Micro-influencer	−0.579	0.012	−0.856	−0.360

## Data Availability

The data that support the findings of this study are available from the corresponding author upon reasonable request.
